# Genome-wide analysis of alternative promoters of human genes using a custom promoter tiling array

**DOI:** 10.1186/1471-2164-9-349

**Published:** 2008-07-25

**Authors:** Gregory AC Singer, Jiejun Wu, Pearlly Yan, Christoph Plass, Tim HM Huang, Ramana V Davuluri

**Affiliations:** 1 Human Cancer Genetics Program, Comprehensive Cancer Center, Department of Molecular Virology, Immunology, and Medical Genetics, The Ohio State University, Columbus, OH, USA; 2 Department of Molecular Genetics, The Ohio State University, Columbus, OH, USA; 3 German Cancer Research Center (DKFZ), Heidelberg, Germany; 4 Center for Systems and Computational Biology, Molecular and Cellular Oncogenesis Program, The Wistar Institute, Philadelphia, PA, USA

## Abstract

**Background:**

Independent lines of evidence suggested that a large fraction of human genes possess multiple promoters driving gene expression from distinct transcription start sites. Understanding which promoter is employed in which cellular context is required to unravel gene regulatory networks within the cell.

**Results:**

We have developed a custom microarray platform that tiles roughly 35,000 alternative putative promoters from nearly 7,000 genes in the human genome. To demonstrate the utility of this array platform, we have analyzed the patterns of promoter usage in 17β-estradiol (E2)-treated and untreated MCF7 cells and show widespread usage of alternative promoters. Most intriguingly, we show that the downstream promoter in E2-sensitive multiple promoter genes tends to be very close to the 3'-terminus of the gene, suggesting exotic mechanisms of expression regulation in these genes.

**Conclusion:**

The usage of alternative promoters greatly multiplies the transcriptional complexity available within the human genome. The fact that many of these promoters are incapable of driving the synthesis of a meaningful protein-encoding transcript further complicates the story.

## Background

**Figure 1:**
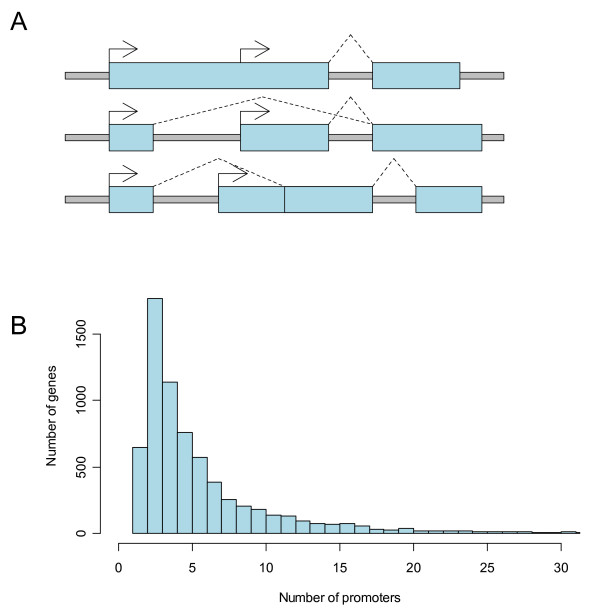
**Alternative promoters can take on several forms (A): Two promoters on a single exon (top); alternative first exons (middle); a downstream promoter is located within the intron region of another isoform (bottom).** The median number of promoters per gene on our microarray is three (B). There are a significant number of single-promoter genes on the array, but these are invariably share a bidirectional promoter with multi-promoter genes.

The regulation of human gene expression is known to be an extraordinarily complex process, including transcription, mRNA processing, mRNA transport, mRNA stability, mRNA translation, protein modification and protein stability. Nevertheless, the picture that has emerged over the past two to three decades is one in which the process of transcription itself is a highly regulated process [1], and one could easily believe that the combinatorial interaction of multiple transcription factors within the gene promoter is sufficient to explain this complexity. However, genes with more than one promoter have been known for some time [2], and recent studies using independent lines of evidence have suggested that a large proportion of the human genome is transcribed from both strands [3] and numerous human genes have more than one promoter allowing gene transcription in different cellular conditions [4-7]. As summarized in Figure 1A, alternative promoters can take many different forms, producing a wide variety of transcripts and proteins from a single gene locus. Moreover, the use of alternative transcription initiation sites also affects the splicing pattern of downstream exons, creating a variety of different transcripts and protein products [8]. It is needless to say that these various promoters greatly increase the regulatory control that the cell has over the expression of the gene.

Alternative promoters are of particular interest because their aberrant expression has been linked to a number of diseases, particularly cancer. There are a number of experimentally well-characterized multiple promoters for known genes, for example *TP53 *[9], *MYC *[10], *CYP19A1 *[11], *BRCA1 *[12], *P73 *[13], *MID1 *[14], *CTSB *[15], *SRC *[16], *KLK6 *[17] and *TGFB3 *[18], to name a few. *CYP19A1 *is a well-characterized example that has five known alternative promoters, many of which are separated by more than 10 kb and are therefore regulated by completely non-overlapping promoters [19]. Alternative first exons Ex-1.1, Ex-1.3/Ex-1.4, and Ex-1f splice with Ex-2 to encode the 5' prime untranslated regions (UTR) of CYP19A1 mRNA in the placenta, adipose tissue, and brain, respectively. Additionally, in gonads, the transcription starts just 39 bp upstream of translation initiation codon in exon-2. The use of alternative non-coding first exons in the CYP19A1 transcripts does not alter the protein sequence, as the different 5'UTRs splice into a common second exon (exon-2) that contains the translation initiation codon. It is known that theses various promoters are used in a tissue-dependent manner [19], but the promoter upstream of exon Ex-1.4 is aberrantly expressed in breast cancer tissue, aggravating the disease [11].

Many putative gene promoters have been identified either through mapping of expressed sequence tags (ESTs) to the genome (Acembly [20], ECGene [21]), through sequence conservation studies with other organisms [22] or *de novo *computational prediction (e.g., FirstEF [23], DragonGSF [24]). Databases such as MPromDb [25] and H-DBAS [26] provide information about well-curated promoters and alternative spliced transcripts identified by aligning completely sequenced and precisely annotated full-length cDNAs [4]. Recently, intensive efforts have been invested in establishing genome-wide profiling methods to identify the regulatory regions, including alternative transcription start sites and the upstream promoter regions in human and mouse genomes [27]. Currently, three ways were applied for this purpose. One is based on the decreased nucleosome occupancy and increased sensitivity to DNase of the active promoter regions. The two approaches, called DNase-chip and DNase-array, have been created to detect those transcribed promoters and transcripts [28,29]. The second one is called the cap analysis gene expression (CAGE), combining full-length cDNA library with SAGE technology to screen those 5' parts of transcripts [30]. The third one is using ChIP-chip (Chromatin ImmunoPrecipitation followed by microarray analysis) to profile the binding position of the RNA polymerase II preinitiation complex [31]. The data from these studies provide evidence of large-scale alternative splicing and wide-spread use of alternative promoters throughout the mammalian genomes. Most of these methods cannot predict the mRNA sequence produced from that promoter, and therefore constructing a traditional cDNA microarray to detect their expression is impossible. Moreover, two promoters may produce mRNA isoforms that are nearly indistinguishable, again making expression microarrays difficult to design. One alternative is to use ChIP-chip to detect the binding of RNA polymerase II to the genome. Although there is evidence that the presence of RNA polymerase II in the promoter does not perfectly correlate with active transcription [32], there does exist a correlation between the two events and therefore RNA polymerase II binding is a good approximation of transcriptional activity [33,34]. Here, we have taken an intermediate approach, where we first annotated all possible putative promoters in the human genome by integrative bioinformatics analyses. Using these annotations, we designed 60-mer probes complementary to sequences and tiling the core promoter regions (both known and putative) of a subset of genes that have at least two annotated promoters. We tested this array by conducting ChIP-chip using antibody against RNA polymerase II (RNA Pol II) in MCF7 cells without and with E2 treatment. It is well known that estrogen receptor can act both as an activator and repressor of specific target genes, and that these events can then affect cell division and breast cancer progression [35,36]. Knowledge about which of the alternative promoters of the ER regulated genes are active and inactive in E2 treated and untreated conditions in MCF7 cells would lead to better understanding of their effects in breast cancer development. Several novel putative promoters were found to be active before and after E2 treatment. Interestingly, we found that in genes with more than one putative promoter, downstream promoters are much more likely to be affected by E2 treatment than upstream promoters, suggesting interesting mechanisms of gene regulation in multiple promoter genes.

## Results

### Alternative promoter array

In order to design an alternative promoter array, we first used a bioinformatics approach to annotate all known and putative promoters in the human genome. Using evidence from three sources: UCSC Known Genes [37], FirstEF [23], and Riken CAGE tags [38], we found evidence for more than 185,000 transcription start sites separated by 500 bases or more in the human genome. We took a gene-centric approach to our microarray design, choosing genes that had two or more known or putative promoters. In the end, about 34,000 known or putative promoters were selected for our array, covering about 7,000 genes. The median number of promoters per gene is three (Figure 1B). 60 mer oligonucleotide probes were designed to tile a region -200 to +200 surrounding each known and putative transcription start site. Because of limitations on probe design, not all regions could be effectively covered but on average the spacing is approximately 80 bases from the end of one probe to the beginning of the next.

### Genome-wide profile of potential promoter usage

**Figure 2:**
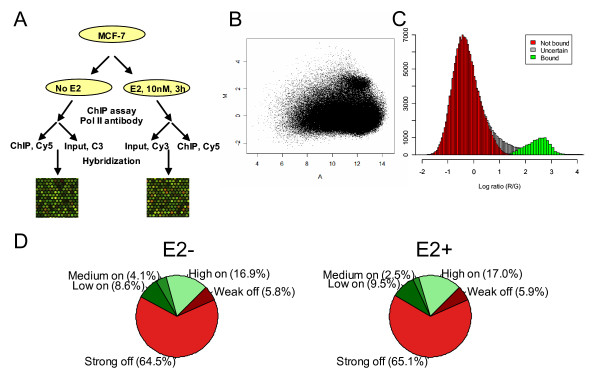
ChIP-chip procedure (A). MA plot for a control experiment, after normalization (B; M = log2(Red/Green); A = log2(Red*Green)/2). Fit of the gamma+normal model to the log ratio of red versus green channels (C). The red portion of the histogram shows probes that belong to the unbound distribution with p < 0.05. The green portion of the histogram are probes that belong to the bound distribution with p < 0.05. The grey areas in between are ambiguous. Our model allows us to annotate promoters as being active or inactive at different confidence levels (D). "High on" indicates strong evidence for RNA Polymerase II binding in both replicates (the probes fall within the green portion of panel C); "Medium on" indicates strong evidence for RNA Polymerase II binding in one replicate, and weak evidence in the other (i.e., the probes fall outside of the red area in panel C). "Low on" indicates weak evidence in both replicates. "Low off" indicates inconsistency between the replicates, and finally "Strong off" indicates a high probability that no binding occurred (i.e., probes fall within the red portion of panel C). "Low on" indicates weak evidence in both replicates. "Low off" indicates inconsistency between the replicates, and finally "Strong off" indicates a high probability that no binding occurred (i.e., probes fall within the red portion of panel C).

In order to identify potential active promoters, we conducted ChIP-chip with antibody against RNA Pol II in MCF-7 cell lines with and without E2 treatment for 3 hours, as described in the Methods. The amplified immunoprecipitated DNA and input control, after labeling with Cy5 and Cy3 fluorescent dyes respectively, was used to probe the alternative promoter microarray (Figure 2A). Each experiment was repeated once to determine the reproducibility of the probe hybridization intensities. After filtering the low quality spots, we performed intensity dependent Lowess normalization. The MA plot for normalized data is shown in Figure 2B for one control (before E2 treatment) experiment. We, then, plotted the distribution of the normalized log ratios of red and green intensities. The histogram in Figure 2C presents the log ratios for one control experiment, which shows a clear bi-modal distribution. The distribution with mode close to zero represents the probes that are non-responsive and the distribution with mode close to 2.5 represents the probes of responsive promoters. The Expectation Maximization (EM) algorithm of Khalili et al [39] was modified from the original Gamma-Normal-Gamma fit to a simple Gamma-Normal fit that appeared to be more appropriate for our data. The algorithm clearly defines two distinct distributions in Figure 2C, representing the unbound probes (in red) and the bound probes (in green). See Additional File 1 for the MA plots and log ratio distribution of data from other experiments. A nice feature of the algorithm is that probes can be assigned to each distribution with a certain probability, allowing us to increase or reduce the stringency of our assignments easily. We defined strong candidates for RNA Polymerase II activity as those probes that fell within the green distribution with a p-value of at most 0.05. However, we also defined a second, weaker condition: those probes that are not significantly part of the larger unbound (red) distribution at a p-value of 0.05. This latter group would encompass the "grey area" that lies between the two distributions. The "best" probe from each promoter was used to evaluate the activity of the promoter as a whole. Figure 2D shows the proportion of active promoters in MCF7 cells at different quality thresholds. At least 65% of the promoters (both putative and known) are inactive in this cell line, whereas ~17% of the promoters have strong evidence for being active. This is roughly in accordance with previous genome-wide studies of promoter activity. For example, Kim *et al *[31] found ~9,300 active promoters in IMR90 cells, which corresponds to ~23% of the unique annotated transcription start sites in the UCSC known genes [37]. When we map these promoters back to genes, we find that 3,210 genes had at least one promoter active in at least on experimental condition, out of a total 6,500 genes for which we were able to recover data – roughly 50%.

**Figure 3:**
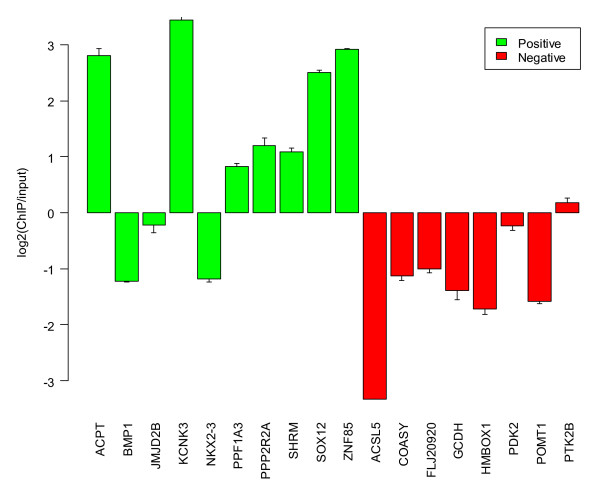
**Seven out of ten promoters were confirmed to be active based on ChIP-PCR assays (green bars).** Similarly, all but one of the promoters called as negative showed no evidence for RNA polymerase II binding (red bars). Error bars indicate standard errors from the mean, based on three replicates.

**Figure 4:**
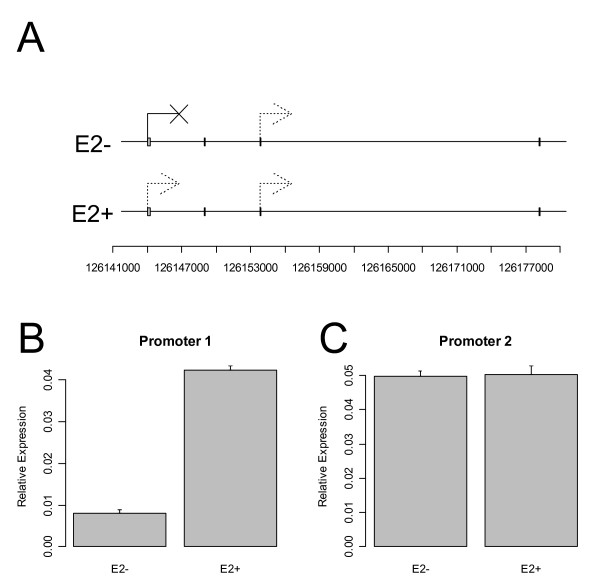
**Shown here are the first four exons of the gene NCOA7, spanning a region of roughly 32 kb (A).** Exon 3 is spliced out of the transcript initiated at Exon 1, but Exon 4 is common to both transcripts. The ChIP-chip microarray analysis indicated that the first promoter is inactive in the control experiment, but is activated with E2 treatment at a low level, a result that is verified by qRT-PCR results (B). The second promoter was predicted to be active at a low level with and without E2 treatment, which again was verified (C). Error bars indicate standard errors from the mean for three replicates.

**Figure 5:**
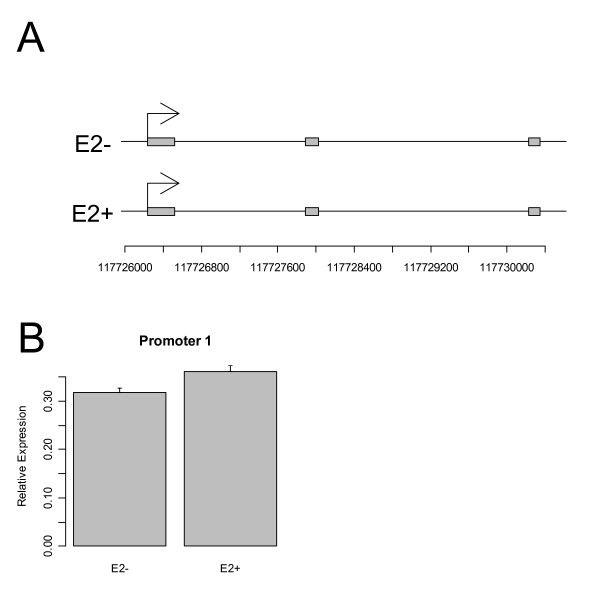
**Shown here are the first three exons of the gene EIF3S9, spanning a region of approximately 3.7 kb (A).** This promoter was shown to be highly active in both treatments, which was verified by qRT-PCR. Error bars indicate standard errors from the mean for three replicates.

We validated a total of 18 promoters, 10 promoters that we predicted to be active with high confidence and 8 promoters that were predicted to be inactive in MCF7 cells. ChIP-PCR experiments showed that these predictions were for the most part accurate (Figure 3) – seven out of the ten positive targets microarray analysis were confirmed to be bound to RNA polymerase II. Similarly, all but one of the negative samples showed no evidence of RNA polymerase II binding. Although the binding of RNA polymerase II to the promoter region needn't correlate to gene expression because of posttranscriptional events, we find that a rough correspondence does exist. For example, two promoters in the gene *NCOA7 *were shown to bind to RNA polymerase II with a "low" level of confidence, although in the absence of E2 the upstream promoter was predicted to be "strongly off" (Figure 4A). These qualitative results were verified by quantitative reverse transcriptase-polymerase chain reaction (qRT-PCR) (Figures 4B and 4C). By comparing these results to the gene *EIF3S9*, whose most upstream promoter was "highly on" in both treatments (Figure 5A), we found that the qRT-PCR experiments showed a correspondingly high level of expression of the corresponding gene isoform (Figure 5B).

### Alternative promoters and CpG islands

Wang *et al*. [40] recently noted that the 5'-most promoter of a gene tends to be CpG related, while more downstream promoters are less likely to be. We identified promoters that were active in one or both of our treatments, and classified them as either being associated with the 5'-end of the gene (if the promoter was located < 500 bases of the 5' end of the gene's annotation) or downstream promoters (> 500 bases away from the 5' end of the gene). Similar to the findings of Wang *et al*., we found that 92% of all 5'-end promoters are associated with a CpG island, whereas only 23% of downstream promoters are.

### Identification of novel promoters

As shown in Table 1, each promoter on the array is supported by different lines of evidence. The most common promoters are those that are supported by multiple CAGE tags. However, only 14% of the 18,902 such promoters supported by only CAGE tags on the array were found to be active at "high" or "medium" confidence levels. Of course, it is important to note that a negative result does not necessarily indicate an inaccurate promoter prediction; these promoters may be active in different cell types, or under different environmental conditions. Therefore, these numbers should be seen as a lower limit. By far, the greatest concordance was found for CpG-related promoters that are supported by all lines of evidence (UCSC Known Genes, CAGE tags, and FirstEF predictions), of these 68% were found to be active. The data also indicate that the CpG-related promoters that are supported by both CAGE tags and FirstEF predictions enjoy a higher rate of success than those promoters that are exclusively supported by either CAGE tags or FirstEF predictions. 16% of non-CpG-related promoters in this category were found to be active, while an impressive 39% of CpG-related promoters supported by CAGE and FirstEF results were found to be active. In all, if we consider all promoters not supported by KnownGenes to be "novel", then out of 20,879 promoters, 3,172 (15%) were active in at least one treatment. If we eliminate promoters supported only by CAGE tags, then 601 out of 1,977 promoters (30%) are found to be active. Of the ten genes selected for validation in Figure 4, eight fall into the novel category (i.e., no mRNA evidence) and six of these were confirmed (see Table 2). These surprising results indicate that large numbers of undiscovered, unannotated promoters exist within human genes. Notably, we have discovered 303 new and active promoters that are situated more than 500 bases upstream of the currently-defined 5' end of the gene, suggesting that a significant fraction of the current gene annotations may not be 5'-complete. One of these promoters was upstream of SOX12, and was verified to bind to RNA polymerase II (Figure 4). These results also strongly support the recent reports of high frequency of alternative promoter in mammalian genomes [41,42]. In addition, the complicated distribution patterns of these alternative promoters might be easily overlooked by previous expression array analyses.

**Table 1:** Activity of promoters with various combinations of supporting evidence

**Evidence**	**CpG-related**	**Number of promoters**	**Number of active promoters**	**Percentage**
KnownGene	Yes	28	2	7.10%
	No	792	84	10.60%
CAGE	Yes	616	38	6.20%
	No	18286	2533	13.90%
FirstEF	Yes	129	26	20.20%
	No	184	48	26.10%
KnownGene, CAGE	Yes	93	9	9.70%
	No	910	81	8.90%
KnownGene, FirstEF	Yes	63	25	39.70%
	No	45	10	22.20%
CAGE, FirstEF	Yes	1123	440	39.20%
	No	541	87	16.10%
KnownGene, CAGE, FirstEF	Yes	3202	2170	67.80%
	No	154	60	39.00%

**Table 2:** Promoters validated by ChIP-PCR and the lines of evidence used to identify them

**Gene Symbol**	**Genomic location (hg18)**	**Evidence**
ACPT	chr19:55989804-55990068	CAGE tags
PPP2R2A	chr8:25959258-25959687	FirstEF
ZNF85	chr19:20897730-20898104	KnownGene
APEG1	chr2:220021650-220021924	FirstEF + CAGE tags
KCNK3	chr2:26804455-26804795	FirstEF + CAGE tags
PPFIA3	chr19:54322857-54323287	FirstEF + CAGE tags
SHRM	chr4:77829597-77830023	FirstEF + CAGE tags
SOX12	chr20:253659-254066	FirstEF + CAGE tags

### Differential use of multiple promoters with estrogen stimulation

**Figure 6:**
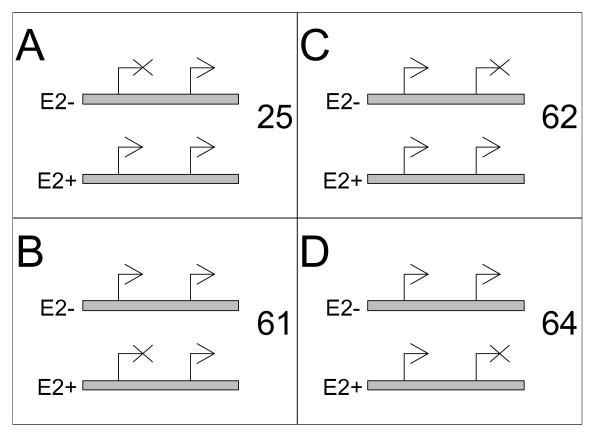
**We found a total of 212 genes that had exactly two promoters that were active in one of these experiments.** Of these, the upstream promoter was activated by E2 in 25 genes (A), and was inactivated by E2 in 61 cases (B). The downstream promoter was activated by E2 treatment in 62 cases (C), and inactivated by E2 in 64 cases (D).

Our hypothesis was that treatment with E2 affects the promoter activity of a sub-set of genes in the genome. For this analysis, we defined "active" as promoters with "high", "medium" or "low" confidence. For the subset of genes that have single active promoter, we found that 2,697 promoters were active in both E2- and E2+ treated conditions (see Additional File 2). Whereas only 178 promoters were inactivated and 77 promoters were activated by E2. This bias towards inactivation is highly significant (p = 2.5e-10 in a chi-squared test), indicating that more promoters are inactivated by E2 than are activated, which supports the previous report about estrogen-mediated early-down regulated genes [43]. Some of the genes associated with these promoters have previously been identified as being estrogen sensitive, such as GREB1, HSPB8, and WFS1 [44] (see Additional Files 2 and 3 for a complete list). We next considered those genes that have two active alternative promoters and checked for the differential activation or inactivation of the promoters. We found 993 genes with both promoters active and not affected by E2 treatment (see Additional File 3). More interesting are the cases where one promoter is affected by E2 treatment. The upstream promoters of 25 such genes are activated by E2 (Figure 6A; also see the gene *NCOA7 *in Figure 4), whereas in 61 genes the upstream promoter is inactivated by E2 (Figure 6B) – a more than 2:1 bias in favor of inactivation, which is quite similar to what we found in the single active promoter gene case, and also significant (p = 0.000175 in a chi-squared test). Curiously, this same bias is not present when we examined the downstream promoters, where we found 62 were activated by E2 (Figure 6C) and 64 were inactivated by E2 (Figure 6D).

**Figure 7:**
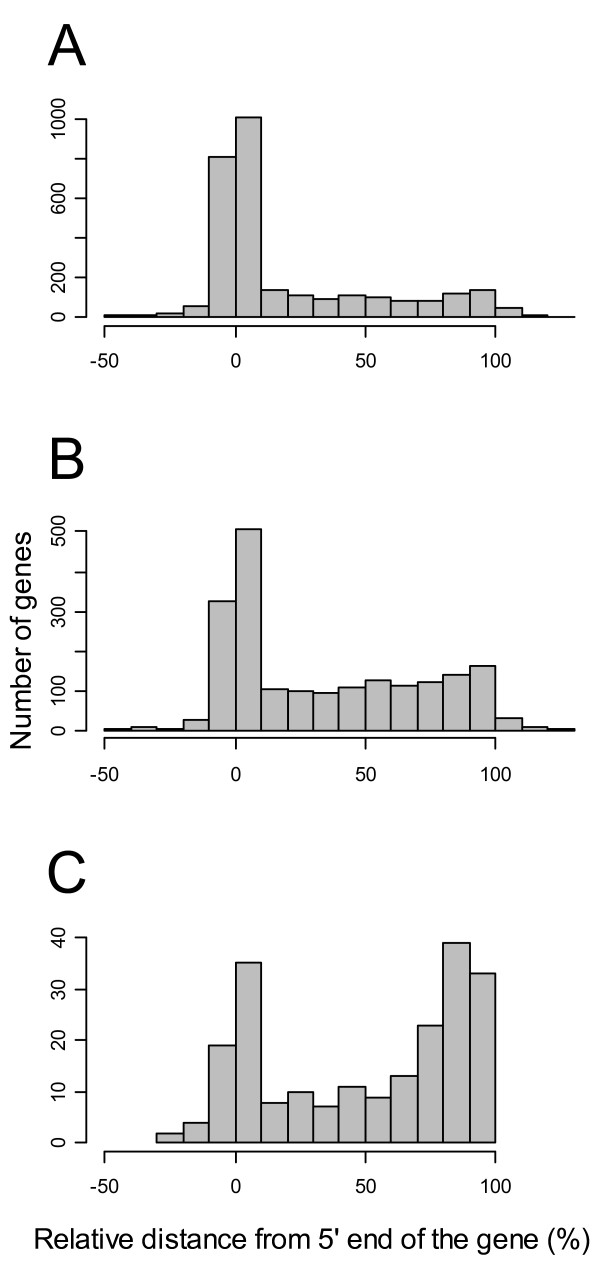
**For genes with a single active promoter, there is a strong tendency for that promoter to be located at the 5' end of the annotated gene, marked as "upstream", versus the 3'-end of the gene which is marked as "downstream" (A).** Similar trends are observed in the case of genes with two active promoters where neither is affected by E2 treatment. Here, one of the promoters is likely to be at the 5' end of the gene, while the other promoter can occur anywhere else along the gene length with roughly equal probability (B). A different pattern is observed in genes with two active promoters where one is affected by E2 treatment (either activated or inactivated). In this case we can see that, as before, the upstream active promoter is likely to be located at the 5' end of the gene, but the downstream promoter is strongly biased towards the 3' end of the gene (C).

In terms of the overall differential usage (either activation or inactivation) of alternative promoters due to E2 treatment, we found that the downstream promoters are more often affected by E2 treatment than the upstream promoter. We found that there were a total of 127 downstream promoters affected by E2 treatment, while only 87 upstream promoters were affected – a significant bias (p = 0.00625 in a chi-squared test). These intriguing patterns provide some insight into the regulatory control of genes and their isoforms by E2. To investigate this phenomenon further, we examined the locations of active promoters within each gene. As shown in Figure 7A, for genes with a single active promoter that is insensitive to E2 treatment there is a strong tendency for that promoter to be located at the 5' end of the annotated gene. Similar trends are observed in genes with two active promoters that are insensitive to E2 treatment, where the upstream promoter is again located near the 5' end of the gene, while the location of the downstream promoter is uniformly distributed throughout the length of the gene (Figure 7B). However, a surprising change is observed if one of the promoters is E2-sensitive, where we found that there was a very strong tendency for the downstream promoter to be close to the 3' end of the gene (Figure 7C). In keeping with our finding that downstream promoters tend to not be associated with CpG islands (in contrast with promoters at the 5'-end of the gene), E2-sensitive promoters are overall less likely to be associated with CpG islands than active promoters taken as a whole: 50% of all active promoters are CpG-related, while only 37% of E2-sensitive promoters are (p = 1.2e-11 in a Fisher's Exact test).

## Discussion

Although genome tiling arrays are increasingly becoming a viable alternative to focused microarrays, they remain significantly more expensive than focused microarrays, and their signal-to-noise ratio is very high due to the large numbers of inactive probes and lack of probe design considerations [45]. Another alternative mechanism for studying alternative promoters is the use of traditional expression arrays that have been designed to specifically interrogate particular gene isoforms. Unfortunately, in a large number of cases, mRNA isoforms are not known for putative promoters, and many isoforms that originate at different promoters differ only in the first exon – a small percentage of the entire molecule, making it difficult to distinguish between the various isoforms. High-throughput sequencing techniques are a recent advance that provide an attractive alternative to microarray-based techniques [33], however there is evidence that ChIP-chip is more sensitive than ChIP-sequencing techniques [46].

Traditionally, expression analysis was used to define promoter activity. However, one recent report has found that a number of genes experience transcription initiation but show not detectable full-length transcripts [32]. Nevertheless, additional reports indicate that a correlation does exist between Pol II occupancy and gene activation [33,34]. The findings of Guenther et al. may be explained by post-transcriptional regulation, but in any case we believe that the presence of Pol II in the promoter is a good approximation of promoter activity, although further experiments are still necessary to define and characterize this relationship.

Here, we have presented a novel 244 k microarray that is capable of measuring alternative promoter usage in over 34,000 putative promoters from nearly 7,000 genes. This platform is suitable for indirect expression analyses using RNA polymerase II ChIP-chip as we have shown in this paper, but it is also suitable for methylation based studies using DMH or meDIP experiments (since more than 5000 of the putative promoters fall within CpG islands), or for ChIP-chip experiments using other proteins of interest, such as transcription factors or histone modification signatures. We have demonstrated clear evidence for alternative promoter activities within genes, including the verification of a number of putative promoters. These results suggest that a large fraction of genes in the human genome possess undiscovered promoters and transcription start sites, which agrees with findings based on the mapping of ESTs to the genome [20,21], and the mapping of 5' oligo cap cDNA libraries to the genome [4].

Most intriguingly, we discovered that there is a distinct bias for the downstream promoter in E2-sensitive two-promoter genes to be very close to the 3' end of the gene, whereas no such bias exists in E2-insensitive genes. These promoters are very unlikely to produce a functional transcript of any sort, and we therefore speculate that its purpose is merely to regulate the expression of the transcript initiated at the upstream promoter by "blocking" the progression of the RNA polymerase II complex. This "stalling" mechanism has been observed in other contexts. For example, inhibiting DNA replication was recently found to cause RNA polymerase II to stall during the transcription of p21 [47]. Similarly, the cofactor of BRCA1 (COBRA1) is known to cause stalling of the RNA polymerase II complex proximal to the promoter [48,49]. However, we can think of no reason for "blocking" promoters to have a bias towards the 3' end of the gene, since this blocking action could be realized at any point relative to the primary promoter. An alternative possibility is that promoters near the 3' end of the gene are driving expression of an interfering RNA, either antisense to the primary transcript or that is capable of inhibiting the formation and progression of the RNA polymerase II complex at the primary promoter [50]. Such noncoding, interfering RNAs are known to regulate expression of the *DHFR *gene in humans, for example, although in this case the interfering RNA is transcribed from a promoter that lies upstream of the primary promoter [51,52]. Much more work will need to be performed in the future to identify the regulatory action that these 3'-UTR promoters have on their primary transcripts, if any.

## Conclusion

We have demonstrated clear evidence of alternative promoter activity for approximately 7,000 human genes, using a 244 K custom microarray that span across 34,000 putative promoters. Our results suggest that a large fraction of genes in the human genome possess undiscovered alternative promoters, which agrees with findings based on the mapping of ESTs and CAGE tags to the human genome. We found that a significantly more number of downstream promoters were affected by E2 treatment than the upstream promoters. And, there is a distinct bias for the downstream promoter in E2-sensitive two-promoter genes to be very close to the 3' end of the gene, whereas no such bias exists in E2-insensitive genes. The custom microarray can also be used for epigenome analyses, such as methylation based studies using DMH or meDIP experiments. The present data will help discovery of novel promoters and ongoing annotation of alternative promoters of human genes in different experimental conditions.

## Methods

### Microarray design

#### Target identification

We considered three sources of evidence for identifying promoter targets for our microarray. The first was the 5'-end of genes as identified in the UCSC Known Gene track, which is largely based on the alignment of RefSeq mRNAs to the human genome [37]. A second line of evidence was the database of CAGE tags sequenced by the Riken group [38]. These tags capture ~20 bases at the 5' end of messenger RNAs, and have been mapped back to the human genome. We used the UCSC LiftOver tool to convert Riken's hg17 human genome coordinates to the more recent hg18 genome. Our final line of evidence was *ab initio *promoter predictions generated by the FirstEF program [23].

Each line of evidence identifies a transcription start site (TSS). We considered TSSs separated by > 500 bp to be distinct promoters – a commonly used criterion. Although there are undoubtedly transcription factor binding sites that extend beyond this region, this distance is great enough for the core promoters of each TSS to be distinct [25], and we can therefore consider these TSSs to be independently regulated to a large extent. TSSs were clustered using a neighbor-joining algorithm [53] until all clusters were separated by at least 500 bases. The coordinates of these clusters were then extended 200 bases up- and downstream.

#### Probe selection

Each promoter region was aligned to the genome using BLAT [54] in order to discover regions that are not unique. Alignments that were longer than 55 bases (90% of the probe length) were masked, as were 60 mers within the sequence that had > 85% or < 50% G+C. From the remaining unmasked regions of each promoter, probes were selected such that the average spacing would be roughly 100 bases, but that the spacing between two successive probes would be no more than 300 bases. In the end, the true average spacing is 80 bases.

#### Gene and promoter selection

Not all genes could be put on the array, so to prioritize we assigned each gene a score. Three points were awarded for each promoter supported by "known gene" evidence, two points for those supported by CAGE tag evidence [38], and one point for FirstEF [23] evidence. Genes were then ranked by their total score, and only the best-scoring genes were included on the array. In the end, the roughly 244,000 probes cover 34,486 promoter regions from 6,949 genes, with a median tiling coverage of 5 probes per promoter. The median number of promoters per gene on the array is 3, although the range is from 1 to over 30 (Figure 1B)

### Cell culture

MCF-7 human breast cancer cells (American Type Culture Collection, Manassas, VA) were maintained in growth medium (MEM with 2 mM L-glutamine, 0.1 mM non-essential amino acids, 50 units/ml penicillin, 50 μg/ml streptomycin, 6 ng/ml insulin, and 10% FBS) as described by Fan et al [55]. Prior to all experiments, cells were cultured in hormone-free basal basal medium (phenol-red free MEM with 2 mM L-glutamine, 0.1 mM non-essential amino acids, 50 units/ml penicillin, 50 μg/ml streptomycin, and 3% charcoal-dextran stripped FBS) for three days.

### Chromatin immunoprecipitation on microarray (ChIP-chip) assay

Five million MCF-7 cells with and without E2 treatment (10 nM, 3 h) were crosslinked with 1% formaldehyde for 10 min, at which point 0.125 M glycine was used to stop the cross-linking. Chromatin immunoprecipitation was performed using a ChIP assay kit (Upstate Biotechnology, Charlottesville, VA) as described [56]. The antibodies, which specifically target against the initiation form of Pol II, were purchased from Santa Cruz Biotechnology (sc-899X, Santa Cruz, CA). Ligation-mediated PCR was applied to 20 ng of ChIP DNA and input control as described by Ren et al [57]. Briefly, after cross-linking, cells were lysed and then sonication was used to shear the chromatin to fragments of around 500 bp. Cell lysis was then subject to immunoprecipitation. After immunoprecipitation, part of supernatant was removed from the lysis as input control. The primers used in ligation-mediated PCR were: oligo JW102, 5'-GCGGTGACCCGGGAGATCTGAATTC-3' and JW103 5'-GAATTCAGATC-3'. Tow μg of amplified ChIP DNA and input control were then labeled by Cy5 and Cy3 fluorescent dyes (Amersham, Buckinghamshire, UK) and were then cohybridized to the custom alternative promoter array. Technical duplication was performed for each sample of ChIP DNA. The slides were washed with three wash buffers (Buffer I. 6× SSPE + 0.005% sarcosine; Buffer 2, 0.06× SSPE; Buffer 3, anti-oxidant mixture in acetonitrile purchased from Agilent) in series at room temperature.

### Chromatin immunoprecipitation- quantitative polymerase chain reaction

ChIP was conducted in the same manner as in the ChIP-chip experiments, described above. The pooled DNA from ChIP and input control were first measured by spectrophotometer (NanoDrop, Wilmington, DE). Quantitative PCR with SYBR green-based detection (Applied Biosystems, Foster City, CA) was performed as described previously. In brief, primers were designed using Primer Express software (Applied Biosystems, Foster City, CA). Quantitative ChIP-PCR values were normalized against values from a standard curve (50 to 0.08 ng, R-squared > 0.99) constructed by input control with the same primer sets.

### Quantitative reverse transcription-polymerase chain reaction

Qiagen RNeasy kit (Valencia, CA) was used to extract total RNA from MCF-7 cells with or without E2 treatment according to the manufacture's manual. Two μg of RNA was first treated with DNase I (Invitrogen, Carlsbad, CA) to remove potential DNA contamination and then was reverse transcribed with SuperScript II reverse transcriptase (Invitrogen, Carlsbad, CA). Quantitative RT-PCR was performed by using SYBR green (Applied Biosystems, Foster City, CA) as a marker for DNA amplification on a 7500 Real-Time PCR System apparatus (Applied Biosystems, Foster City, CA). The relative mRNA level of a given locus was calculated by relative quantization of gene expression (Applied Biosystems, Foster City, CA) with glucose phosphate isomerase mRNA as an internal control.

### Microarray analysis

The washed slides were scanned by a GenePix 4000A scanner (Axon, Union City, CA) and the acquired microarray images were analyzed using GenePix 6.0 software. Briefly, the user-selectable laser power settings for Cy5 (635 nm, red) and Cy3 (532 nm, green) were adjusted so that the overall Cy5 to Cy3 ratios were close to 1 and that the signal intensities spanned the entire spectrum with minimal signal saturation at the high intensity range. When these conditions were satisfied, the microarray was scanned and a grid file was loaded to mark the general location of the scanned image. The GenePix 6.0 software performed a spot finding function and captured intensity-related information in a GPR file.

The complete array dataset can be viewed in the ArrayExpress microarray database (accession number E-MEXP-1644). GPR files were passed through a custom-built quality control filter which flagged all probes that didn't meet all of the following criteria in both the green and red channels: (1) % > B + 2SD greater than 30; (2) median – background > 0; (3) signal-to-noise ratio greater than 1.5. These filtered results were then normalized using the default parameters (plus Lowess normalization) in Agilent's Chip Analytics software version 1.3. A post-normalization MA plot is shown in Figure 2B. We then used a modified version of the mixture model (reducing their gamma+normal+gamma model to a simple gamma+normal model) described by Khalili et al [39] to classify probes into one of two groups: bound or not bound. Figure 2C shows the fit of the gamma+normal mixture model to our data. One benefit of this type of analysis is that we are able to directly estimate our false positive rates based on a probe's probability of assignment to the "unbound" distribution.

Since each promoter contains many probes, for each promoter region we chose the probe with the best p-value for inclusion into the "bound" distribution and compared these across the various experimental treatments and replicates. We found that between replicates these "best probes" were within 80 bases of each other (the resolution of the array) 90% of the time. We used the following criteria to classify promoters within individual experiments: strongly bound promoters had probes that were classified in the "bound" distribution with a p-value less than 0.05. Weakly bound promoters were those that did not significantly fall within the "unbound" distribution with a p-value of 0.05. Unbound promoters were those whose probes fell within the "unbound" distribution with a p-value less than 0.05. As Figure 2D illustrates, by combining replicate experiments, we were able to classify each promoter into "highly on" (both replicates were strongly bound), "medium on" (one replicate was strongly bound, the other weakly bound), "low on" (both replicates are weakly bound), "weakly off" (replicates don't agree, so we fall back on the null hypothesis of no binding), or "strongly off" (both replicates show an unbound state).

## Abbreviations

TSS: transcription start site; ChIP-chip: Chromatin Immunoprecipitation (ChIP) followed by microarray analysis; E2: 17β-estradiol; CAGE: cap analysis gene expression; RNA Pol II: RNA polymerase II.

## Authors' contributions

GACS designed the computational methods and performed the statistical analyses. JW designed the experimental methods and performed the ChIP-chip experiments. PY coordinated the microarray experiments. CP participated in the design of the study. RVD and THMH formulated and directed the design of the study. All authors read and approved the final manuscript.

## Supplementary Material

Additional File 1 M-A plots for the four ChIP-chip experiments. MA plots for the two control and two E2 treated experiments, data after normalization (B; M = log2(Red/Green); A = log2(Red*Green)/2). The plots show two distinct clusters of points. The larger cluster of probes represents those that are not bound to RNA polymerase II, while the smaller cluster (higher on the M axis) represents bound probes. Click here for file

Additional File 2 List of gene promoters active in both control (E2-) and E2 treated (E2+) conditions. Column 1 shows the Gene symbol, column2 gives the genomic location of the promoter (hg18 build); columns 3 and 4 show the activity of the promoter (1 – active and 0 – inactive) in E2- and E2+ conditions. Click here for file

Additional File 3 List of genes with 2 promoters, both active and not affected by E2 treatment. Column 1 shows the Gene symbol, columns 2 and 3 give the genomic location of the alternative promoters – promoter 1 and promoter 2 (hg18 build); columns 4,5,6 and 7 show the activity of the two promoters (1 – active and 0 – inactive) in E2- and E2+ conditions. Click here for file
