# Supplementary figures and images for: Genome-wide analysis of alternative promoters of human genes using a custom promoter tiling array
Source: BMC Genomics. 2008 Jul 25;9:349. doi: 10.1186/1471-2164-9-349 (PMC2527337; doi:10.1186/1471-2164-9-349)

**Control-1**

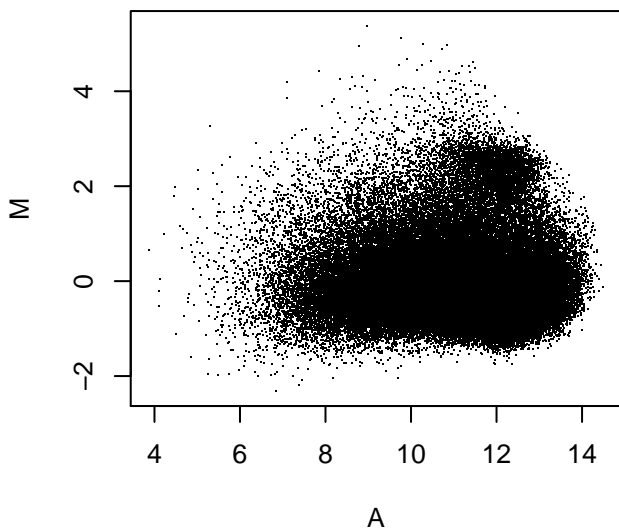

**Control-2**

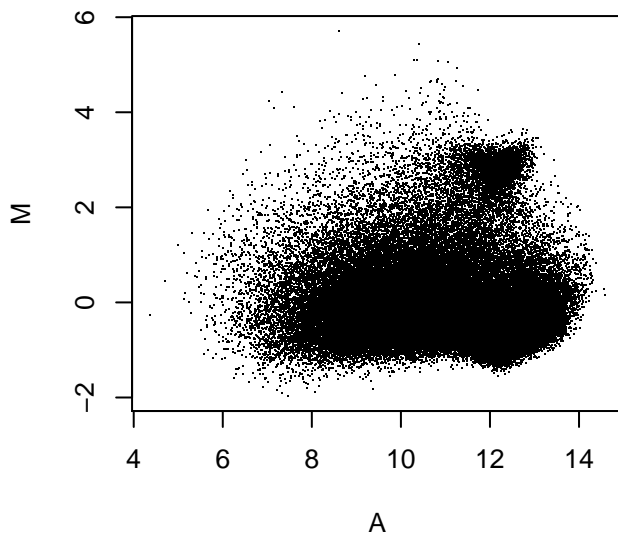

**E2-1**

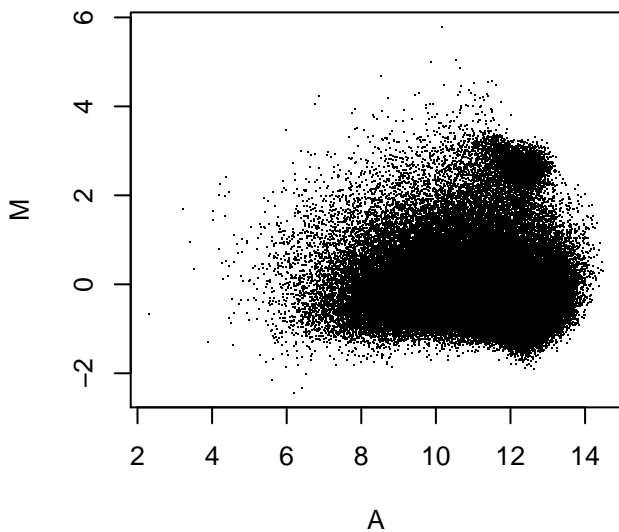

**E2-2**

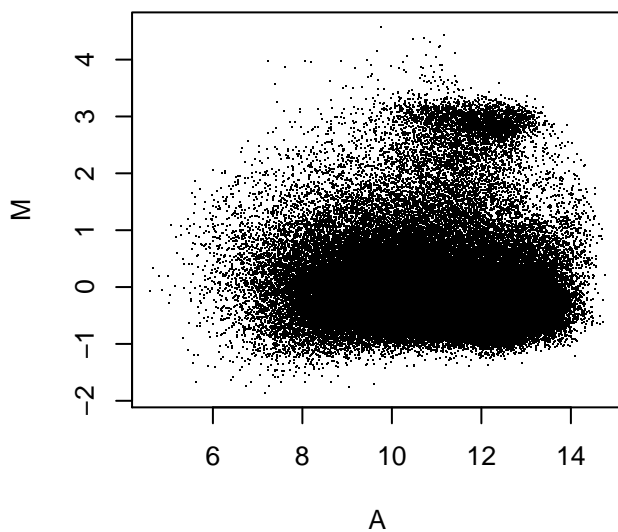

Supplement: Additional File 1 — M-A plots for the four ChIP-chip experiments. MA plots for the two control and two E2 treated experiments, data after normalization (B; M = log2(Red/Green); A = log2(Red*Green)/2). The plots show two distinct clusters of points. The larger cluster of probes represents those that are not bound to RNA polymerase II, while the smaller cluster (higher on the M axis) represents bound probes. [file 1471-2164-9-349-S1.pdf]
